# Single tunnel technique versus coracoid sling technique for arthroscopic treatment of acute acromioclavicular joint dislocation

**DOI:** 10.1038/s41598-022-07644-z

**Published:** 2022-03-10

**Authors:** Liangquan Peng, Yizi Zheng, Siyu Chen, Shiwei Yang, Junjie Liu, Chao Cheng, Greg Zhang, Zhenhan Deng

**Affiliations:** 1 Department of Sports Medicine, The First Affiliated Hospital of Shenzhen University, Shenzhen Second People’s Hospital, Shenzhen, 518035 Guangdong China; 2 Guangxi University of Chinese Medicine, Nanning, 530229 Guangxi China; 3 Department of Thyroid and Breast Surgery, Shenzhen Breast Tumor Research Center for Diagnosis and Treatment, National Standardization Center for Breast Cancer Diagnosis and Treatment, The First Affiliated Hospital of Shenzhen University, Shenzhen Second People’s Hospital, Shenzhen, 518035 Guangdong China; 4 Teaching Office, Shenzhen Second People’s Hospital, Shenzhen, 518035 Guangdong China; 5 Department of Orthopedics, Shenzhen Shekou People’s Hospital, Shenzhen, 518067 Guangdong China; 6 Department of Orthopaedics, Yiyang Central Hospital, Yiyang, 413000 Hunan China; 7 McGovern Medical School, University of Texas Health Science Center at Houston, Houston, TX 77054 USA

**Keywords:** Trauma, Medical research

## Abstract

To evaluate and compare the efficacy of two techniques for the treatment of acute acromioclavicular joint dislocation, the charts of 60 patients diagnosed with acute Rockwood type IV and V acromioclavicular joint dislocation that undergone arthroscopic fixation procedure with single tunnel technique (N = 30, 30.7 ± 5.7 years old) or coracoid sling technique (N = 30, 30.1 ± 5.4 years old) fixation were retrospectively reviewed. The Visual Analog Scale pain score, Constant shoulder functionality score, Karlsson acromioclavicular joint score, the time of return to sports and activity, and plain radiographs of the affected shoulder at different time points of follow-up were recorded for a minimum of 2 years post-op. The majority of the patients recovered to their preoperative activity levels with few complications. The average postoperative acromioclavicular and coracoclavicular distances were significantly narrower than preoperative measurements in both groups without significant difference between the two groups at 2 years post-op (P < 0.05). The coracoid sling technique group had reduced operative time, shorter time of recovery of shoulder movements, higher Constant functionality scores and Karlsson acromioclavicular joint scores, and fewer complications than the single tunnel technique group at the last follow-up (P < 0.05). Therefore, coracoid sling technique achieved superior clinical outcomes with fewer complications compared to the traditional single tunnel technique in arthroscopic treatment of acute acromioclavicular joint dislocation.

## Introduction

Acromioclavicular (AC) joint injury accounts for approximately 9% to 12% of all traumatic shoulder girdle injuries^1^. One in three of these shoulder injuries show radiological evidence of broadening or dislocation of the AC joint. Overall, young men were at higher risk, and more than half of these injuries related to exercise^2^. The Rockwood classification distinguishes 6 total grades of the severity of acute lesions and is the most commonly used system to guide treatment decision-making^3^. Surgical indications are still controversial, but a common consensus has been reached between the so-called “low-energy” and “high-energy” trauma patterns^4^. It is generally agreed that type I-II injuries are caused by low-energy trauma and should be treated conservatively using a sling or harness, whereas type IV-VI dislocations are caused by high-energy trauma and require surgical treatment. However, the treatment of type III lesions is still a controversial issue: some clinicians recommend surgical treatment, while others advocate for non-operative treatment.

More than 150 variations in surgical methods have been reported for the treatment of symptomatic AC joint dislocation so far^5^. These various options range from rigid fixation with Kirschner wires, tension bands, or hook plates, to the Weaver-Dunn procedure, which is a ligament reconstruction procedure based on the transference of AC ligament^6^. However, there is no consensus on the optimal surgical technique. Arthroscopy can better and more clearly show the situation around the coracoid; in addition, there is no need for extensive dissection of the deltotrapezial fascia. The clearer visibility also reduces the risk of damage to important adjacent neurovascular structures^7^. Previous works have also reported good curative effects by using the arthroscopic TightRope system or paired Endobutton Technique (PET) for surgical treatment of acute AC joint dislocation.

Among these methods, the single tunnel technique (STT) using the TightRope system or PET is the most widely utilized^8,9^. However, a bone tunnel that is drilled through the clavicle to the coracoid process (CP) is the key procedure in this technique, which requires accurate localization in order to reduce the risk of fracture of CP. Coracoid fractures remain a significant complication that occur predominately in techniques utilizing bone tunnel^10^. Milewski et al. found a 20% incidence of coracoid fractures in the coracoid tunnel group in their series of 27 patients undergoing a resuspensory reconstruction for high-grade acromioclavicular joint injuries^11^. Coracoid fractures can be symptomatic or asymptomatic, but may result in failures of the operation^12–14^. During an arthroscopically assisted procedures, it is very difficult to judge first-pass accuracy of tunnel placement. Therefore, it has been accentuated that transclavicular-transcoracoid drilling should be approached with caution^15^.

In order to solve this problem, we have created a new technique using the TightRope system or PET to fix the AC joint without creating the bone tunnel to CP. The purpose of this retrospective study is to analyze the clinical data and radiographic findings of the coracoid sling technique (CST) and compare its outcomes with that of STT. We speculate that fixation of the AC joint dislocation using CST will provide stable fixation and satisfactory clinical function.

## Methods

### Patients

The retrospective study was performed with the approval of the Ethics Committee and carried out in accordance with the Helsinki Declaration. All patients provided signed preoperative informed consent. Charts of patients with acute AC joint dislocation undergoing AC joint fixation with STT or CST between June 2009 to June 2018 at our institution were reviewed. Patient data were collected retrospectively through the electronic medical record system. The clinical variables of interest include patient’s gender, age at surgery, injury mechanism, Rockwood classification of injury, and surgical technique. All the study participants claimed preoperative shoulder pain and weakness interfering with their activities of daily living. Typical symptoms include the following: pain over the AC joint, a feeling of AC joint instability with popping or grinding sensation, shoulder muscle fatigue, as well as shoulder deformity.

The inclusion criteria were as follows: (1) acute (injury less than 2 weeks) AC dislocation (Rockwood type IV or V); (2) age range from 18 to 45 years; (3) without osteoporosis (bone mineral density was tested by dual energy X-ray absorption method and osteoporosis was defined as T ≤ -2.5SD); (4) all surgeries performed by the same surgeon; (5) over 2 years follow-up. Exclusion criteria: (1) existing history of bone disease; (2) previous shoulder surgery; (3) open and old dislocations; (4) complicated by associated injuries, such as nerve or vascular injury, fractures, or dislocation of other parts of the ipsilateral limb.

Radiographs of the bilateral shoulder joints in anteroposterior (AP) and lateral scapular (Y) positions were taken in every patient preoperatively.

### Surgical techniques

Patients were in the beach-chair position with the administration of an interscalene nerve block combined with general anesthesia. Intraoperative equipments included anterior cruciate ligament (ACL) tip-to-tip tibial aimer (Smith & Nephew, MA, USA), paired EndoButton device (Smith & Nephew, MA, USA) or TightRope system (Arthrex, Naples, FL, USA), and high strength wires (Ultrabraide, Smith & Nephew, MA, USA).

**Figure 1:**
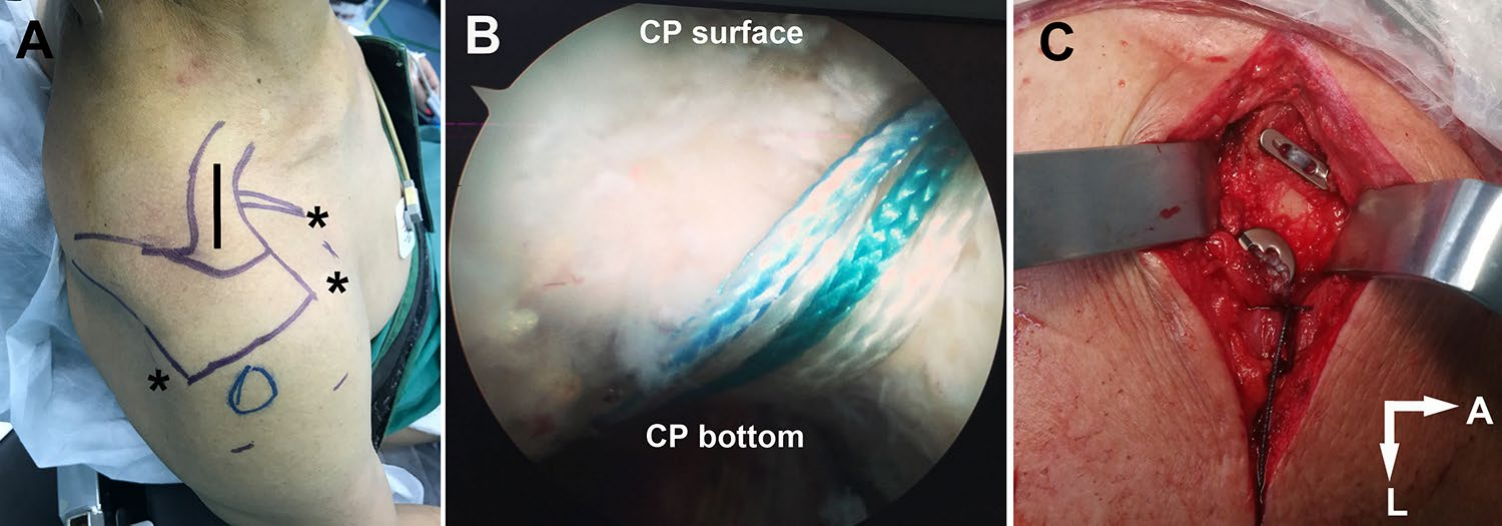
CST surgery for treatment of the AC joint dislocation. (**A**) Three portals (mark as “*”, standard posterior portal, anterior portal, and anterolateral portal) and one 2-cm transverse incision (mark as “-”) directly over the AC joint were made on the skin. (**B**) The non-absorbable sutures were hung at the bottom of the CP under arthroscopy. (**C**) TightRope fixation system being placed on the surface of clavicle.

The similar procedure had been described in our previous publication^16^. To begin, the standard posterior inspective portal and anterolateral portal were created. Radiofrequency dissector was used to dissect the anterior capsule over the subscapularis tendon. The lower surface of the subcoracoid was completely debrided in order to clearly expose the CP base. Then, a 2 cm incision was established over the AC joint to expose the distal clavicle (Fig. 1A). AC articular disc was resected when it hampered AC joint reduction. Furthermore, the distal clavicle was excised 3–4 mm in order to achieve good reduction. A 2.4 mm Kirschner wire (K-wire) was used to fix it temporarily.

### Single tunnel technique (STT)

The tip of the ACL tibial aimer was placed at the center of the CP base. The targeting tip was placed at the superior surface of the clavicle 2–3 cm medial to the AC joint line and 5 mm anterior to the rear border of the clavicle. Next, drill a 2.4 mm guide pin from the clavicle down directly in line with the base of CP, and use a 4.5 mm reamer to form a tunnel through the clavicle and CP. A 2–0 polydioxanone suture (PDS) was used as a guiding suture and penetrate the bone tunnel from clavicle to the CP. One Endobutton was pulled through the clavicle and CP tunnel and secured against the inferior cortex of CP, while another Endobutton was placed on the surface of the clavicle. After further reducing the coracoclavicular (CC) distance, the non-absorbable sutures on the Endobutton button were tightened and cinched.

### Coracoid sling technique (CST)

**Figure 2:**
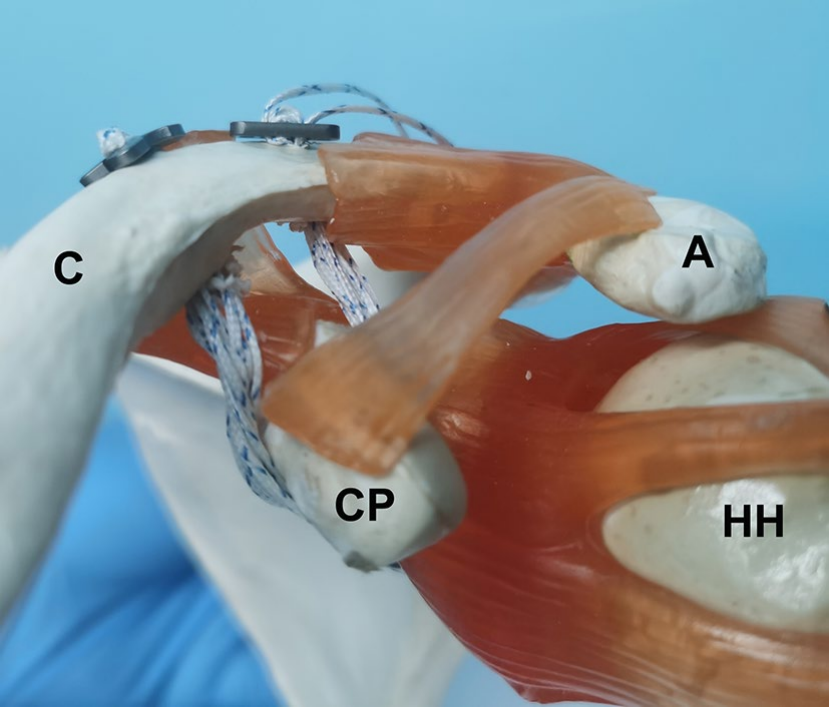
The principle of the CST surgery is shown in a plastic shoulder joint model. A: acromion. C: clavicle. CP: coracoid process. HH: humeral head.

Firstly, two bone tunnels were established at 1.5 cm (lateral tunnel) and 3.5 cm (medial tunnel) to the distal clavicle with a 4.5 mm reamer. Insert the PDS guide line through the medial tunnel and directed to the medial side of the CP base, bypassing the lateral side of the CP base, and going through the lateral tunnel from the bottom up. One Endobutton was pulled into the medial tunnel, bypassing the CP base, and pulled out from the lateral tunnel. Another Endobutton was fixed at the socket of the medial tunnel, and the previous Endobutton was stuck at the lateral tunnel socket. After further reduction of the CC distance, The non-absorbable sutures on the Endobutton were tightened and cinched (Fig. 1B,C). The principle behind the CST surgery is shown in a plastic shoulder joint model (Fig. 2).

Following fixation of the paired EndoButton device/TightRope system with any of the two aforementioned techniques, the CC ligament was restored with the high strength wires. Finally, the K-wire was removed, the ruptured capsule was sutured, and skin was closed. Here, there is no need to remove the Endobutton. The operative period of the two techniques were recorded and compared.

### Rehabilitation protocol

The same rehabilitation program was utilized in both groups. The shoulder joint was placed in a sling sponge shoulder abduction immobilizer at 0° external rotation position for 6 weeks post-op. From the second postoperative day onwards, passive Gentle pendulums and Codman’s were encouraged depending on the patients’ pain tolerance level. Patients were instructed to not resume active movement of the arm until 6 weeks post-op. Patients were generally allowed to return to normal activities and daily work but limited sports until 3 months post-op depending on the level of rehabilitation. In addition, they were asked not to engage in contact sports prior to six months post-op.

### Follow-up

Patients were followed-up at the outpatient setting at 3, 6, 12, and 24-months post-op. The following subjective and objective outcomes were recorded at each follow-up visit: Visual analog scale (VAS) for pain (0: no pain; 10: worst possible pain), Shoulder Constant score (100: no pain; 0: maximum pain), the time of return to normal activities and sports, self-reported symptoms and complications, as well as the Karlsson ACJ score (Grades A–C)^17^.

The CC distance (vertical distance between the inferior border of the clavicle and superior border of the CP), and the AC distance (vertical distance between the superior edge of the acromion to the superior edge of the distal clavicle) in both groups were determined via standard AP position X-ray radiographs, and radiographic analysis was presented in millimeters (mm) and compared preoperatively and at 2 years postoperatively^18^. For the contralateral side, an increase in CC distance by 50–100% and increase in CC distance higher than 100% were considered to be subluxation and redislocation, respectively^19^.

### Statistical analysis

The measured data was expressed as mean ± SD and analyzed statistically using SPSS software (version 18.0; SPSS, Chicago, IL). The paired t-test was used for pairwise comparison, and SNK q test was used for comparison between multiple groups. All tests were carried out within a 95% confidence interval in which p < 0.05 was considered statistically significant. Intra-observer and inter-observe reproducibility were evaluate by intraclass correlation coefficient (ICC). ICC less than 0.4 means poor reliability, greater than 0.75 means good reliability.

## Results

### Baseline characteristics

Table 1 tabulates the characteristics of the two groups of patients. A total of 90 patients who underwent arthroscopic acute AC joint dislocation fixation was selected. There was no statistically significant difference in age, body mass index (BMI), gender ratio, affected side, cause of injury, Rockwood classification, interval between injury to operation, or length of follow-up among the patient groups (p > 0.05). All cases need to be followed up for at least 2 years before they can be included in the study.

**Table 1:** Demographic characteristics of the two groups at baseline.

Parameter	STT	CST
Age (years)	30.7 ± 5.7	30.1 ± 5.4
Body mass index (BMI)	24.1 ± 3.0	23.3 ± 3.1
**Gender**		
Male	21	23
Female	9	7
**The affected side**		
Left	10	12
Right	20	18
**Cause of injury**		
Sports	17	15
Motor vehicle accident	9	10
Fall	4	5
**Rockwood classification**		
Type IV	9	12
Type V	21	18
Interval between injury to operation (days)	8.1 ± 3.4	8.7 ± 3.1
Length of follow up (months)	25.3 ± 1.7	25.6 ± 2.1

### AC and CC distances measurement

**Figure 3:**
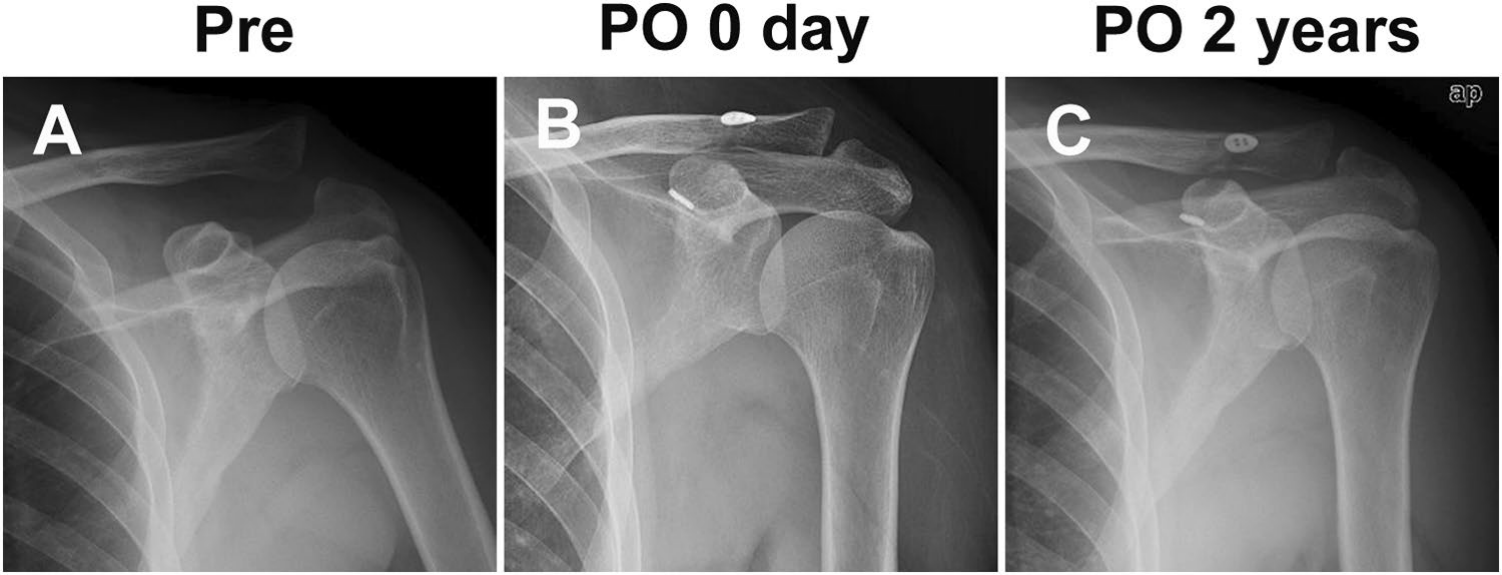
Pre- and post-operative AP view of an AC joint dislocation using STT surgery. (**A**) AC joint dislocation preoperatively. (**B**) Restoration of AC joint immediately after CST surgery. (**C**) Restoration of AC joint at 2 years following CST surgery.

**Figure 4:**
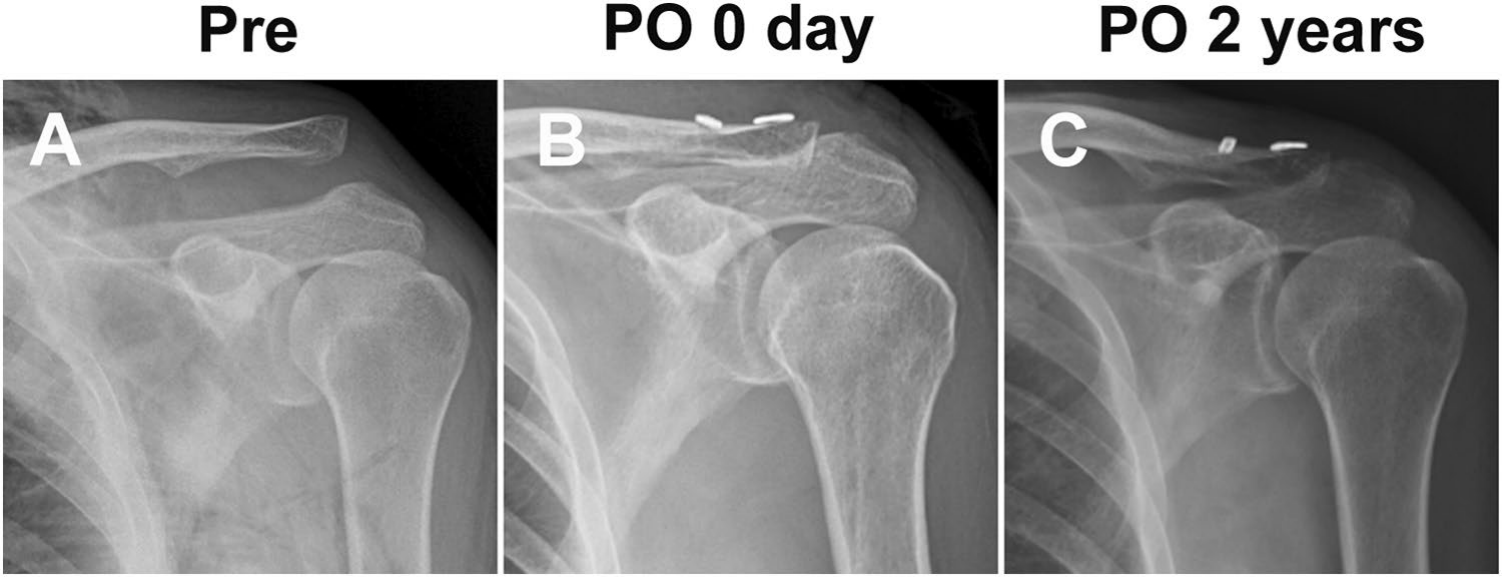
Pre- and post-operative AP view of an AC joint dislocation using CST surgery. (**A**) AC joint dislocation preoperatively. (**B**) Restoration of AC joint immediately after STT surgery. (**C**) Restoration of AC joint at 2 years following STT surgery.

**Figure 5:**
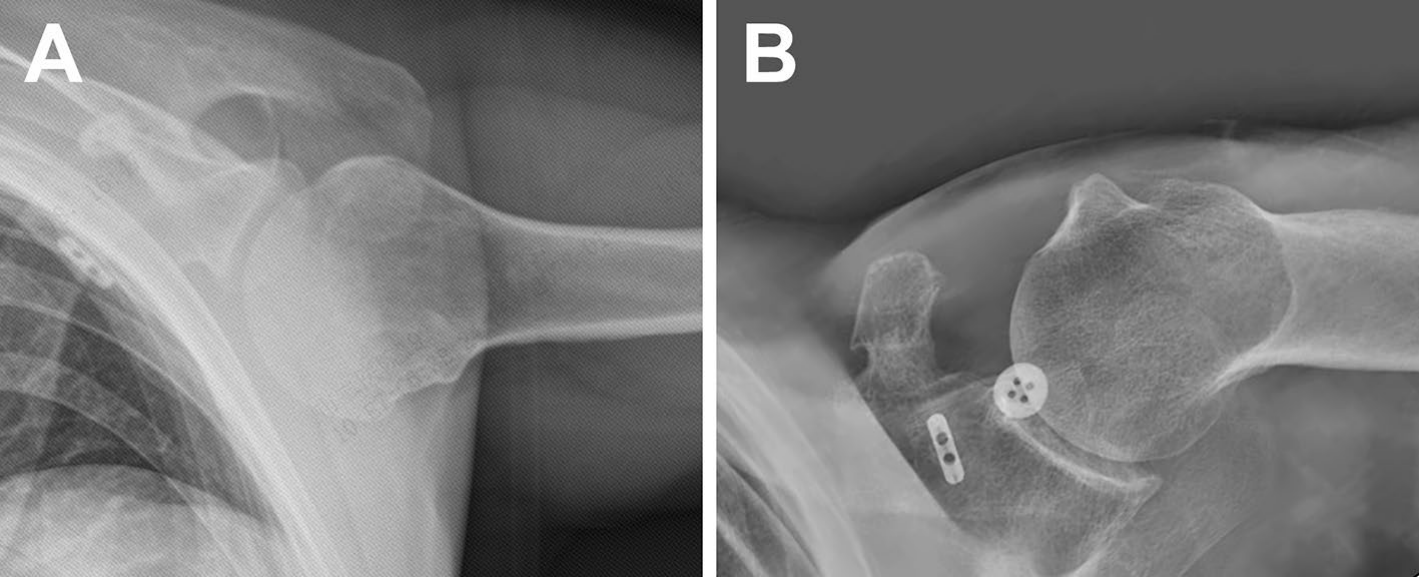
Post-operative axillary view of AC joint dislocation using STT (**A**) or CST (**B**) surgeries at the last follow-up.

Dislocation of the AC joint was found in both groups preoperatively. During the operation, all the dislocated AC joints achieved completely reduction. In several cases, the reductio was hampered by twisted or locked articular disc and the articular disc was removed. No one performed distal clavicle resection. Radiographic examination showed that the AC joint was completely reduced immediately post-op (PO 0 days) and remained stable at 2 years after operation (Fig. 3, 4, 5).

The intra-observer reproducibility was 0.991 (95%CI 0.987 ~ 0.993), and the inter-observer reproducibility was 0.989 (95%CI 0.986 ~ 0.993). At baseline, there was no significant difference in the average AC and CC distances of the injury joints between the two groups preoperatively (p > 0.05, Table 2). At the time point of follow-up for 2 years, the average AC and CC distances was still significantly narrower than before surgery in both groups (p < 0.01). Furthermore, two years after operation, there was no significant difference in the average distance of AC and CC between the two groups (p > 0.05). In addition, at the final 2 years follow-up time point, there was no significant difference in average AC and CC distances compared with the normal side shoulder joints in both groups (p > 0.05).

**Table 2:** Comparison of AC and CC distance in the both groups measured from preoperative and 2 years postoperative radiographs.

Group	AC distance			CC distance		
	Preoperative(NS)	Preoperative(IS)	Postoperative(IS)	Preoperative(IS)	Postoperative(IS)	Preoperative(NS)
STT	1.6 ± 0.6	5.9 ± 0.9	1.8 ± 0.5*	13.9 ± 2.5	8.2 ± 1.4	7.7 ± 1.2
CST	1.5 ± 0.5	6.1 ± 1.0	1.7 ± 0.4*	14.2 ± 2.3	7.9 ± 1.3	7.5 ± 1.3

Note: IS: injury shoulder, NS: normal shoulder; *P < 0.05 compared with injury side preoperatively; # P < 0.05 compared with normal side preoperatively; ✠P < 0.05 comparison of the CST and the STT group.

### Clinical outcomes

We were delighted to find that the operation time in the CST group was significantly shorter than that in the STT group (96.3 ± 14.3 vs 73.7 ± 17.3 min, p < 0.01, Table 3). At the final follow-up, most of the patients in both groups were firmly fixed and fully recovered.

**Table 3:** Evaluation Results of Two Fixation techniques.

Parameters	Preoperation		Postoperation		p value
	STT	CST	STT	CST	
Operation time (min)	**–**	**–**	96.3 ± 14.3	73.7 ± 17.3	< 0.01
Cases return to former sports	**–**	**–**	19(63.3%)	25(83.3%)	< 0.01
Time of return to sports (mon)	**–**	**–**	5.1 ± 1.3	4.2 ± 1.4	< 0.01
VAS score	7.8 ± 1.6	7.7 ± 1.5	1.8 ± 1.3	1.7 ± 1.2	> 0.05
Constant score	24.1 ± 2.4	23.5 ± 3.2	81.7 ± 3.6	90.2 ± 4.1	< 0.01
**Karlsson**					
A	**–**	**–**	19	25	< 0.01
B	**–**	**–**	7	4	
C	**–**	**–**	4	1	
Total Complications	**–**	**–**	7(23.3%)	2(6.7%)	< 0.01
Loss of reduction	**–**	**–**	2(6.7%)	0	< 0.01
Redislocation	**–**	**–**	3(10.0%)	0	< 0.01
Infection	**–**	**–**	2(6.7%)	2(6.7%)	> 0.05

In the CST group, 25 patients returned to the previous levels of exercise and activity at an average time of 4.2 months post-op, while 5 patients were unable to return to their previous level of activity. In the STT group, 19 patients resumed their previous sports and activities at an average of 5.1 months post-op, while 11 patients were unable to return to their previous level of activity, indicating that patients treated with CST needed a shorter interval to recover their previous activity levels than those treated with STT (p < 0.01, Table 3).

At 2 years post-op, no significant difference in VAS scores were detected between the two groups (p > 0.05, Table 3). Nevertheless, the CST group had shorter time to recovery of shoulder movements, in addition to higher Constant functional scores and Karlsson AC joint scores than the STT group (p < 0.01). Therefore, compared to the STT group, the CST group achieved better clinical outcomes.

### Complications

In the majority of the cases upon reviewing the postoperative radiographs, especially in the CST group, the paired EndoButtons or TightRope systems were properly placed. However, complications did occur in several cases during the follow-up time period. In the STT group, there were two loss of reduction cases as the Endobutton on the CP side slipped out, but satisfactory outcomes were still achieved after revision using the previous technique. Three patients were determined to have Rockwood type II dislocation at their last follow-up. Of these cases, two were eroded by Endobutton into the cortex of clavicle or CP, and one was caused by the separation of the Endobutton. Two cases of infection were observed and all recovered completely after medical treatment. There were also 2 cases of postoperative infection in the CST group. one recovered completely after conservative treatment. Another reported reinfection at 4 months after operation, and antibiotic treatment was ineffective. Consequently, we removed the pair of Endobuttons and did not observe any sign of redislocation during the remining follow-up period. There were no cases of neurovascular damage or post-traumatic arthritis of the injured AC joint in both groups.

## Discussion

The CC ligaments originated from the superior-posterior direction of CP, and inserting into the lateral-inferior side of the clavicle. It is a complex composed of two separate ligaments, namely conoid ligament (medial aspect) and trapezoid ligament (lateral aspect). As a vertical stabilizer of the AC joint, when the CC ligaments stretch in response to clavicle rotation, it allows for 20º of movement of the shoulder. The CC ligaments act as a suspender for the clavicle and scapula. When the CC ligament is ruptured post trauma, the biomechanical balance of the surrounding structures is disrupted. The traction force from the sternocleidomastoid muscle will result in posterior upper shifting of the clavicle as well as separation of the AC joint^20^. The most common method of AC joint dislocation is to repair and strengthen CC ligament. Endobuttons are placed on the clavicle and CP and are subsequently connected with loop, non-absorbable sutures with the same function of the native CC ligaments, fixing the AC joint and restoring CC ligaments, which are prerequisites for biological fixation^21^. At the same time, suturing and repairing the AC ligaments during surgery, restoring the horizontal stability of AC joint, preserving the deltotrapezial fascia, and beneficial to the restoration of AC stability.

The mechanical strength of the fixation device represented by Endobutton was proved to be better than that of autologous tendon ^22^. The Tightrope system consists of one round clavicle titanium button and one long coracoid titanium button connected by non-absorbable sutures (No. 5 Ethibond suture), was initially used to treat acute syndesmosis disruption. The paired Endobuttons device tightened with high strength wires have a similar function. Their scope of application has been extended to treat AC joint dislocations^23^. The Tightrope system represents a promising method for stabilizing acute AC joint separation^24,25^, not only in higher grades of AC dislocations (type IV and V), but also in type III dislocations^26^.

All the patients in this study were acute AC joint dislocations. The fixation methods that used could achieve good reduction effect and the ruptured CC ligament could heal, Therefore, ligament reconstruction was not required. The establishment of the bone tunnel from clavicle to CP is the key step in the SST. Usually, a tip-to-tip aimer would be applied for positioning purposes. However, at present, some orthopaedic departments, specially that of basic-level hospitals, have not provided this instrument, which limits the application of this technology. STT is technically difficult and has an increased risk of fracture, and is sometimes extremely challenging in patients with a small CP, which is an irregular structure with a relatively thin cortex^27,28^. Whether it is the correct location or the accurate drilling of an accurate bone tunnel requires a great deal of experience, which is a great challenge for arthroscopists^24^. The excessive tension of the paired Endobutton might increase the risk of Endobutton slippage from the surface of clavicle and CP, especially when the Endobutton is placed on the uneven bone surface of the clavicle or the CP. Furthermore, the pressure of the Endobuttons on the cortex of clavicle and CP may be too concentrated, leading to chronic bone erosion and eventually resulting in AC joint laxity^29,30^. In the cases treated with SST technique, these complications caused by intraosseous button displacement are mainly due to excessively broad bone tunnel and limited Endobutton plate area^31^.

In contrast, CST requires fewer skills and is less time-consuming. CST requires fewer steps; most significantly, it eliminates the need to drill the bone tunnel in CP, significantly reducing the tunnel related complications. In the CST group, We did not observe any cases of button slippage, erosion, or AC joint instability. This technique does not require special tools; therefore, it can be widely promoted in orthopaedic surgery departments in lower level of care settings. TightRope Endobutton restores the conoid profile of the CC ligaments in an roughly anatomically correct position and reconstructs the bio-mechanical stability of the joint. The TightRope provides long-term stability for the joint, and its strength and stiffness are better than those of natural CC ligaments. At the same time, fixation with TightRope can not only maintain the mircomotion of AC joint, but also promote immediate stability, early shoulder exercise, and improved recovery^32^. In our study, patients were instructed to start rehabilitation exercise on the first postoperative day, and we found fast recovery times and high fraction of patients resuming their previous activity levels in the CST group. In addition, our results show that there is no significant difference in AC and CC distances between the two groups at 2 years follow-up, indicating both techniques could ensure rigid fixation. Most importantly, our study patients treated with CST surgery were superior in functional outcomes as they achieved higher Constant functional scores and Karlsson AC joint scores than the STT group did.

Despite the advantages shown, this study also presents some limitations. First of all, the cases enrolled in this study were not as many as we had desired, and the postoperative follow-up period is relatively short. In order to draw more definitive conclusions, we will increase and analyze more cases of simultaneous use of these two surgical techniques, and follow-up in future work. Secondly, the pressure of Endobuttons exerted on bone surface and the tension of sutures on CP was not measured. In order to solve this problem, systematic biomechanical tests will be carried out to reveal the mechanism. Thirdly, no pre- or post-operative weighted bilateral view was obtained. Though the functional outcomes were significantly elevated, the radiographic results may be overestimated. Fourthly, our previous study had compared single and double TightRope systems ^16^, However, double TightRope technique was not compared in this study. We will perform study focused on the comparison between these techniques in order to better reveal the pros and cons of CST. Lastly, biomechanical study suggested that an additional AC cerclage is needed to adequately reconstruct physiological horizontal stability of the AC joint ^33^. However, this procedure was not performed in our cases. We will add AC cerclage in our future works in order to achieve better stabilization.

In conclusion, the presented study proved that application of CST technique achieved better clinical outcomes with fewer complications in arthroscopic treatment of in acute AC joint dislocation compared with traditional STT technique; this technique could be considered a reliable method for AC joint reconstruction.

### Ethics approval

This study was approved by the Ethical Committee of the First Affiliated Hospital of Shenzhen University, Shenzhen Second People’s Hospital. A written informed consent was obtained from the patients for the publication of this study and any accompanying images.

### Consent to participate

A written informed consent was obtained from the patients for the publication of this study and any accompanying images.

## Data Availability

All data generated or analysed during this study are included in this published article.
